# Nuclear Transport Modulation Reduces Hypercholesterolemia, Atherosclerosis, and Fatty Liver

**DOI:** 10.1161/JAHA.113.000093

**Published:** 2013-04-24

**Authors:** Yan Liu, Amy S. Major, Jozef Zienkiewicz, Curtis L. Gabriel, Ruth Ann Veach, Daniel J. Moore, Robert D. Collins, Jacek Hawiger

**Affiliations:** 1 Department of Medicine, Division of Allergy, Pulmonary and Critical Care Medicine, Vanderbilt University School ofMedicine, Nashville, TN (Y.L., J.Z., R.A.V., J.H.); 2 Division of Cardiovascular Medicine, Vanderbilt University School of Medicine, Nashville, TN (A.S.M.); 3 Department of Pediatrics, Ian Burr Division of Endocrinology and Diabetes, Vanderbilt University School of Medicine, Nashville, TN (D.J.M.); 4 Department of Pathology, Microbiology, and Immunology, Vanderbilt University School of Medicine, Nashville, TN (A.S.M., C.L.G., R.D.C.); 5 Department of Molecular Physiology and Biophysics, Vanderbilt University School of Medicine, Nashville, TN (J.H.)

**Keywords:** atherosclerosis, cell‐penetrating peptides, fatty liver, hypercholesterolemia, hypertriglyceridemia, importins, inflammation, karyopherins, nuclear signaling, sterol regulatory element binding proteins, transcription factors

## Abstract

**Background:**

Elevated cholesterol and triglycerides in blood lead to atherosclerosis and fatty liver, contributing to rising cardiovascular and hepatobiliary morbidity and mortality worldwide.

**Methods and Results:**

A cell‐penetrating nuclear transport modifier (NTM) reduced hyperlipidemia, atherosclerosis, and fatty liver in low‐density lipoprotein receptor‐deficient mice fed a Western diet. NTM treatment led to lower cholesterol and triglyceride levels in blood compared with control animals (36% and 53%, respectively; *P*<0.005) and liver (41% and 34%, respectively; *P*<0.05) after 8 weeks. Atherosclerosis was reduced by 63% (*P*<0.0005), and liver function improved compared with saline‐treated controls. In addition, fasting blood glucose levels were reduced from 209 to 138 mg/dL (*P*<0.005), and body weight gain was ameliorated (*P*<0.005) in NTM‐treated mice, although food intake remained the same as that in control animals. The NTM used in this study, cSN50.1 peptide, is known to modulate nuclear transport of stress‐responsive transcription factors such as nuclear factor kappa B, the master regulator of inflammation. This NTM has now been demonstrated to also modulate nuclear transport of sterol regulatory element‐binding protein (SREBP) transcription factors, the master regulators of cholesterol, triglyceride, and fatty acid synthesis. NTM‐modulated translocation of SREBPs to the nucleus was associated with attenuated transactivation of their cognate genes that contribute to hyperlipidemia.

**Conclusions:**

Two‐pronged control of inflammation and dyslipidemia by modulating nuclear transport of their critical regulators offers a new approach to comprehensive amelioration of hyperlipidemia, atherosclerosis, fatty liver, and their potential complications.

## Introduction

Hyperlipidemia is a risk factor in cardiovascular and hepatobiliary morbidity and mortality worldwide.^1^ In the United States, this risk places a huge burden on the estimated 33 million Americans with hypercholesterolemia, taking an annual death toll of >800 000 from cardiovascular disease.^2^ Excessive accumulation of cholesterol and triglycerides leads to atherosclerosis in lipid‐laden blood vessels and steatosis in the liver.^3^ We hypothesized that modulating nuclear transport of proinflammatory transcription factors would regulate these inflammatory responses to metabolic insults and ameliorate atherosclerosis and steatohepatitis. To test this hypothesis, we applied cell‐penetrating cSN50.1 peptide, a nuclear transport modifier (NTM), to the model of accelerated atherosclerosis in low‐density lipoprotein (LDL) receptor‐deficient (*ldlr*^*−/−*^) mice. NTMs (SN50, cSN50, and cSN50.1) have been proven effective in modulating nuclear transport of proinflammatory stress‐responsive transcription factors (SRTFs) by competing with their binding to importins alpha.^4–6^ Surprisingly, we found that cSN50.1 not only modulates nuclear transport of SRTFs such as the transcription factor nuclear factor kappa B (NFκB), but also sterol regulatory element‐binding protein (SREBP) transcription factors that regulate lipid homeostasis.

The cardinal role for SREBPs in maintaining and/or altering lipid homeostasis is compellingly documented in genetic experiments. More than 30 genes that encode cholesterol‐ and triglyceride‐synthesizing enzymes and binding proteins are regulated by SREBPs, and their excessive nuclear transport is associated with deranged lipid homeostasis.^7–9^ Overexpression of nuclear forms of SREBP‐1a, SREBP‐1c, and SREBP2 in transgenic mice led to striking phenotypes of a 28‐fold increase in cholesterol synthesis and massive fatty liver.^7,10–11^ In contrast, SREBP1 deficiency in the model of accelerated atherosclerosis in *ldlr*^*−/−*^ mice prevented Western diet–induced hyperlipidemia and mitigated atherosclerosis.^12^ However, the content of cholesterol and triglycerides in the liver was not reduced.

SREBPs lack a nuclear localization sequence (NLS) for binding to importins alpha. Nuclear import of SREBPs is mediated by binding to importin beta instead.^13^ We discovered that cSN50.1 interacts with importin beta, and reduces nuclear translocation of SREBP1 and SREBP2 induced by lipid depletion in cultured cells. Therefore we examined the effect of treatment with NTM on hypercholesterolemia, hypertriglyceridemia, atherosclerosis, body weight gain, fatty liver, and hyperglycemia in *ldlr*^*−/−*^ mice fed a Western diet high in fat and cholesterol.

## Methods

### Preparation of NTM Peptides and Peptide Modules

Cell‐penetrating peptides SN50 (2780 Da) and cSN50.1 (2986 Da) and the peptide modules listed in Table 1 were synthesized by standard solid‐phase peptide synthesis using Fmoc chemistry with an automated peptide synthesizer. A double‐coupling cycle was used for less reactive amino acids: arginine, lysine, glutamine, aspartic acid, and glutamic acid. Biotin was added in a double‐coupling cycle (3 hours each) at the end of synthesis using standard coupling reagents to produce tagged peptides for pull‐down assays, yielding ≈65% biotinylation of peptide chains, as determined by high‐performance liquid chromatography (HPLC). A hydrophilic 5 or 7 amino acid tag was added to Signal Sequence Hydrophobic Region (SSHR) peptide modules to facilitate solubility. Each crude peptide was precipitated as a TFA salt in cold ethyl ether and then purified by HPLC on a modified semipreparative C18 reverse‐phase column. The main fractions were combined, solvent removed by SpeedVac concentration, then lyophilized, and the final product was stored desiccated at 4°C.

**Table 1.** Amino Acid Sequences of NTM Peptides* and Peptide Modules

	SSHR	NLS
SN50	AAVALLPAVLLALLAP	VQRKRQKLMP
cSN50.1	AAVALLPAVLLALLAP	CVQRKRQKLMPC
N50		VQRKRQKLMP
cN50.1		CVQRKRQKLMPC
SSHR‐1*	AAVALLP	
SSHR‐2*	AVLLALLAP	

Hydrophobic regions of the SSHR domain are distinguished from the cluster of basic amino acids (NLS). Combinations of listed peptides were tested in pull‐down assays. Because of limited solubility of SSHR‐derived peptides in aqueous solution, a solubility tag was added to the amino terminus of these peptides. For pull‐down experiments, all peptides were labeled with biotin. NTM indicates nuclear transport modifier; SSHR, signal sequence hydrophobic region; NLS, nuclear localization sequence.

* N‐biotinylated versions of these peptides were used in binding and competition assays.

* Solubility tag added to the N‐terminus of these peptides.

### Mouse Studies of Hyperlipidemia, Atherosclerosis, and Fatty Liver

All animal experiments were carried out in strict accordance with the recommendations in the Guide for the Care and Use of Laboratory Animals of the National Institutes of Health, and protocols were approved by the Vanderbilt University Institutional Animal Care and Use Committee. Six‐week‐old B6.129S7‐Ldlrtm1Her/J female mice (*ldlr*^*−/−*^*)* were purchased from Jackson Laboratories. This strain of mice develop elevated serum cholesterol and triglyceride levels, increased liver cholesterol, and develop atherosclerotic lesions and liver inflammation when fed a high‐fat diet.^14^ Mice were fed a Western diet containing 21% milk fat and 0.15% cholesterol for up to 8 weeks, and both mice and food were weighed at the beginning and end of each experiment. Mice were treated with cSN50.1 peptide beginning at the onset of diet change, except as indicated (n≥5 per experimental point or condition as indicated). Age‐matched controls received sterile saline in the same volumes as cSN50.1. For intraperitoneal administration, 200‐μL aliquots containing 0.4 or 0.7 mg of cSN50.1 in saline were injected at 8‐ or 12‐hour intervals as indicated. Alternatively, 10 mg of cSN50.1 in 100 μL of sterile H_2_O was administered subcutaneously from ALZET Osmotic Pumps (1007D) placed aseptically in interscapular areas. A ketamine/xylazine cocktail (70 mg/kg ketamine+13 mg/kg xylazine IP) was used for anesthesia to immobilize the mice for pump placement. A bolus of 0.7 mg of cSN50.1 in 100 μL of saline was administered intraperitoneally at weekly pump changes to assure steady cSN50.1 bioavailability. Dosage schedules were developed on the basis of previous experimental protocols^6,15–16^ and half‐life studies.^5,17^ Feces were collected 1 day before euthanization, and food was removed the night before mice were euthanized. Blood was collected at the time of euthanization, and fasting chemistries were determined in mouse plasma using an automated chemistry analyzer in the Vanderbilt Clinical Research Center. Due to the limitations of collecting samples repeatedly from the same animal, results obtained at different time points represented separate groups of animals. Total cholesterol and triglycerides in liver and cholesterol in feces were analyzed by standard methods in the Lipid Core Laboratory.^18–19^ Complete blood cell counts were performed in the Clinical Hematology Laboratory, and flow cytometry analysis of lymphocyte subsets was conducted as previously described.^20^ Cryostat sections of livers were stained with Oil‐red‐O. Atherosclerotic lesions were analyzed in the aortic root by staining with Oil‐red‐O.^21^ Mice were perfused with saline, and hearts were removed, embedded in OCT, and frozen on dry ice. Frozen blocks were stored at −20°C until sectioning. To obtain sections of the aortic sinus, hearts were cut until the 3 leaflets of the sinus were visible. At that point every other 10‐μm section was collected to total of 15 sections/sinus or a total of 300 μm of the aortic sinus. Sections were baked overnight at 55°C and stained the next day for neutral lipids using Oil‐red‐O and counterstained to visualize nuclei with hematoxylin. The average Oil‐red‐O staining area was measured (excluding the internal elastic lamina) using Image Pro analysis software by averaging all fifteen 10‐μm sections/mouse.

### Importins Pull‐Down Assay

Whole‐cell extracts of unstimulated human Jurkat T cells or serum‐starved human HepG2 cells were prepared by lysing cells in binding buffer (10 mmol/L HEPES at pH 7.9, 150 mmol/L NaCl, 10 mmol/L KCl, 2.5 mmol/L MgCl_2_, 1 mmol/L EGTA, 1 mmol/L DTT, 0.1% NP‐40, 1% protease inhibitor cocktail). Cell lines were obtained from the American Type Culture Collection. Ten nanomoles of biotinylated NTM peptides or their constituent modules depicted in Table 1 (SN50, cSN50.1, N50, cN50.1, SSHR‐1, SSHR‐2) were incubated overnight with 1.5 mg of whole‐cell extract at 4°C. The mixture was then cleared by centrifugation (16 000*g* for 30 minutes at 4°C) and supernatant transferred to a fresh tube containing high‐capacity Neutravidin beads (Thermo Scientific) to bind biotinylated peptides. After 4 hours, beads were separated from the mixture by centrifugation (30 seconds at 1000*g*) and washed 3 times with binding buffer and 2 times with PBS. Beads were boiled for 10 minutes in SDS loading buffer, and protein content was analyzed by immunoblotting with a panel of antibodies to importins alpha and beta (see below) and GAPDH (Abcam) as a cellular protein control.

### Nuclear Transport Modifier/Importin Beta Competition Binding Assay

Nonbiotinylated SN50 peptide (at 0, 10, 30, 100, or 300 μmol/L concentrations) was incubated overnight with 1 mg of human Jurkat T cell whole‐cell extract at 4°C. Simultaneously, biotinylated SN50 peptide (10 nmol) was immobilized on high‐capacity Neutravidin beads at 4°C overnight, and then beads were washed 3 times with PBS and 1 time with binding buffer to remove unbound biotinylated SN50. The mixture of cell extract and nonbiotinylated peptide was precleared by centrifugation (16 000*g* for 30 minutes at 4°C) and supernatant transferred to a fresh tube containing immobilized biotinylated SN50 peptide. Immunoblot analysis of importin beta bound to beads was performed as described in the importins pull‐down assay.

### Human Hepatoma (HepG2) Cell Culture and Treatment Protocols

HepG2 cells were maintained in DMEM supplemented with 10% FBS, 10 mmol/L HEPES, penicillin (100 U/mL), and streptomycin (100 μg/mL). Before cell extracts were prepared for importins pull‐down assays, HepG2 cells were starved overnight in DMEM containing 1% FBS. For rapid sterol depletion, cell monolayers were rinsed with Hank's buffered saline (HBSS), then incubated in DMEM supplemented with 5% delipidized FBS (Tissue Culture Biologicals; 1 mg/dL cholesterol), 10 mmol/L HEPES, penicillin (100 U/mL), streptomycin (100 μg/mL), and 1% hydroxypropyl‐β‐cyclodextrin (HPCD; MP Biomedicals) for 15 minutes. Monolayers were then rinsed in the same medium without HPCD and incubated in the same medium without HPCD but containing 10 μmol/L MG132 and with 0, 10, or 30 μmol/L cSN50.1 for 2 hours. For gradual sterol depletion, cell monolayers were rinsed with HBSS, then incubated in DMEM supplemented with 5% delipidized FBS, 10 mmol/L HEPES, penicillin (100 U/mL), and streptomycin (100 μg/mL) with 0, 10, or 30 μmol/L cSN50.1 for 24 hours. MG132 (10 μmol/L) was added 2 hours before harvest. For glucose activation, HEPG2 cells were starved for 24 hours in DMEM containing 5.5 mmol/L glucose, then refed with 25 mmol/L glucose+ 100 nmol/L insulin±30 μmol/L cSN50.1 for 24 hours.

### Preparation and Immunoblot Analysis of Liver and HepG2 Cell Extracts

Whole‐cell extracts were prepared from snap‐frozen livers by homogenization of liver pieces (100 mg) in ice‐cold RIPA buffer supplemented with protease and phosphatase inhibitors and 1% NP‐40. Homogenates were centrifuged at 10 000*g* for 30 minutes at 4°C and supernatants used for immunoblotting. Nuclear and cytosolic extracts were prepared from frozen livers or HepG2 cells as previously described^22^ with the addition of phosphatase inhibitors. Liver pieces were disrupted in a Dounce hand homogenizer on ice without NP‐40 and cells pelleted at 4000*g* for 1 minute before extract preparation. Extracts were analyzed by quantitative immunoblotting using monoclonal anti‐SREBP1 (2A4, Novus) and antiphosphorylated NFκB p65 (93H1; Cell Signaling) or polyclonal anti‐SREBP1, anti‐SREBP2, anti‐NFκB p65, anti‐NPC1L1, anti‐HDAC3 (Santa Cruz), anti‐SREBP2 (Thermo), and anti‐ChREBP (Novus) on a Licor's Odyssey Infrared Imaging System.^23^ Polyclonal anti‐GAPDH (Abcam) or monoclonal anti‐beta actin (AC15; Abcam) were used as loading controls for normalization in cytosolic and whole‐cell extracts, whereas polyclonal anti‐Lamin A/C (Cell Signaling) and anti‐Lamin B (Santa Cruz) were used as loading controls for normalization in nuclear extracts as indicated. All lanes shown in each row are from the same membrane for each primary antibody, and the same membrane was blotted for the appropriate loading control protein. When multiple antibodies were used for immunoblotting of the same samples on different membranes, a representative membrane showing the loading control protein is displayed. For quantitative analyses, all membranes were immunoblotted for the loading control protein. The positions of pSREBP and nSREBP bands in immunoblots of liver extracts were verified by immunoblotting with antibodies preincubated with a 5× molar excess of antibody‐specific blocking peptides.

### Quantitative PCR

To quantify the expression of mRNA involved in cholesterol homeostasis, total RNA was isolated from livers of saline‐ and peptide‐treated mice, and cDNA was synthesized using an iScript cDNA Synthesis Kit (Bio‐Rad). The assay mix was made by combining cDNA, Taqman Gene Expression Master Mix (Applied Biosystems), and Taqman gene expression probes for indicated genes according to the manufacturer's instructions. The data were analyzed using SDS2.3 software, and the Ct values were calculated using RQ Manager Software (Applied Biosystems). All transcript levels were normalized to 18S mRNA, and values are expressed as 2^−ΔΔCt^ with the average of the saline‐treated control group serving as the comparator.

### Statistical Analyses

Statistical comparisons between groups were performed with GraphPad Prism 6. Data are expressed as the mean + SEM, or ±SD as indicated in figure legends. Data collected from animal samples did not have normal distribution; therefore, nonparametric tests were chosen. For analysis of atherosclerosis data in Figure 2A and 2D, we chose the non‐parametric Kruskal–Wallis test with a Dunn's Multiple Comparison posttest to correct for multiple comparisons and compare the mean rank of each treatment group column with the mean rank of the saline control column using significance without confidence intervals. All other data collected from animals were analyzed using a non‐parametric unpaired Mann–Whitney test. For analysis of immunoblotting data from cultured cells, the unpaired Student's *t* test with Welch's correction was employed, except for analysis of concentration effects where ordinary one‐way ANOVA with a Tukey's multiple comparison post test was used to compare the mean of each column with the mean of every other column using confidence intervals and significance. For all analyses, *P<*0.05 was considered significant.

## Results

### NTM–Treated *ldlr*^*−/−*^ Mice Fed a Western Diet Have Lower Levels of Cholesterol, Triglycerides, and Glucose in Plasma and Gain Less Weight Than Saline‐Treated Controls

We targeted nuclear transport in vivo in atherosclerosis‐prone *ldlr*^*−/−*^ mice fed a Western diet by treating them with the NTM cSN50.1 peptide (Table 1). NTMs are first‐in‐class 26 or 28 amino acid fragment‐linked peptides derived from the signal sequence hydrophobic region (SSHR) of human fibroblast growth factor 4 and from the NLS of human nuclear factor kappa B 1. The SSHR enables facile intracellular delivery of cell‐penetrating peptides to modulate nuclear transport of SRTFs in circulating blood cells as well as organs.^24^ By occupying NLS‐binding pockets on importins alpha, NTMs prevent their interaction with SRTFs that encompass nuclear factor kappa B (NFκB), activator protein‐1 (AP‐1), nuclear factor of activated T cells (NFAT), signal transducer and activator of transcription 1 (STAT1), and NF‐E2‐related 2 (Nrf2); see Table 2.^4–5,4–26^ In the nucleus, SRTFs activate a myriad of genes that encode mediators of inflammation; NTM suppresses this process in cultured cells and in vivo.^6,16,26,28–29^ The cyclized form of NTM used in this study, cSN50.1, is a cell‐penetrating peptide that is highly soluble (100 mg/mL) and easily delivered to blood cells, liver, spleen, pancreas, and lungs. NTMs protect these organs from microbial and autoimmune inflammation, including type 1 diabetes.^15–16,20,29^ Initially, in pilot experiments to test the effect of NTM on hypercholesterolemia, there were 2 control groups, animals treated with either a non‐cell‐penetrating peptide composed of only the NLS (cN50.1) diluted in saline as a vehicle or saline alone. The results of the experimental end points analyzed below did not differ significantly between these 2 control groups (not shown). Therefore, subsequent experiments were conducted with control animals receiving saline only.

**Table 2.** List of Transcription Factors Known to Be Modulated by Nuclear Transport Modifiers (SN50, cSN50, cSN50.1)

Transcription Factor*	Length (AA)	UniProt	Reference	Function
NFκB1 (p105 or p50)	p105: 968 p50: 433	P19838	^4^	NF‐κB (nuclear factor kappa–light‐chain enhancer of activated B cells) transcription factor is a hetero‐ or homodimeric protein complex that controls transcription of >200 genes in response to stimuli such as stress, cytokines, free radicals, ultraviolet irradiation, and oxidized low‐density lipoprotein (LDL), as well as bacterial and viral antigens. NF‐κB regulates the immune response. Upregulation of NF‐κB has been linked to inflammatory and autoimmune diseases, sepsis, viral infections, disorders of immunity, and cancer, in which antiapoptotic pathways prevail in contrast to inflammation‐driven apoptosis in certain cell types.
Rel A (p65)	551	Q04206	^4^	
Rel B	579	Q01201	^25^	
c‐Rel	619	Q04864	^4^	
NFκB2 (p100 or p52)	p100: 900 p52: 454	Q00653	^25^	
AP‐1 (cFos/cJun)	c‐Fos: 380 c‐Jun: 331	P01100 P05412	^5^	AP‐1 (activator protein 1) transcription factor is a heterodimeric protein composed of c‐Fos and c‐Jun. It regulates gene expression in response to a variety of stimuli, including cytokines, growth factors, stress, and bacterial and viral infections. AP‐1 regulates cell differentiation, proliferation, and apoptosis. In some cells AP‐1 plays a proapoptotic role by regulating the expression of a specific subset of target genes that promote efficient apoptotic cell death following mitotic arrest.
NFATc (NFATc1)	943	O95644	^5^	NFAT (nuclear factor of activated T cells) constitutes a family of transcription factors shown to be important in immune response. NFAT is also involved in the development of the cardiac, skeletal muscle, and nervous systems. The NFAT transcription factor family consists of 5 protein members: NFATc1, NFATc2, NFATc3, NFATc4, and NFAT5.
STAT1	750	P42224	^5^	STAT (signal transducer and activator of transcription) transcription factor regulates cell growth, differentiation, and survival. There are 7 mammalian family members: STAT1, STAT2, STAT3, STAT4, STAT5 (STAT5A and STAT5B), and STAT6. Dysregulation of these proteins leads to increased angiogenesis, enhanced survival of tumors, and immunosuppression. STAT proteins are also involved in the development and function of the immune system and play a role in maintaining immune tolerance and tumor surveillance.
Nrf2	605	Q16236	^26^	Nrf2 (nuclear factor [erythroid‐derived 2]‐like 2) transcription factor is also known as NFE2L2. Nrf2 is essential for inflammasome activation and exacerbates atherosclerosis without altering lipid metabolism (see reference ^26^ in the text). Nrf2 increases the expression of several antioxidant enzymes.
SREBP1	Precursor: 1147 Nuclear: 490	P36956	This study	SREBP (sterol regulatory element‐binding proteins) transcription factors are required for lipid homeostasis. They regulate transcription of >30 known genes that encode participants in the cholesterol, triglyceride, and fatty acid synthetic and uptake pathways. SREBPs belong to the basic‐helix–loop–helix leucine zipper class of transcription factors. Low levels of sterols induce their cleavage, forming a water‐soluble N‐terminal domain containing a bHLH‐Zip motif that is translocated to the nucleus. There are 2 distinct isoforms of SREBP1: 1a and 1c. Although SREBP‐1a regulates genes related to lipid and cholesterol synthesis and its activity is regulated by sterol levels, SREBP‐1c regulates genes required for glucose metabolism and fatty acid and lipid production. Expression of SREBP‐1c is regulated by insulin.
SREBP2	Precursor: 1141 Nuclear: 484	Q12772	This study	
ChREBP	852	Q9NP71	This study	ChREBP (carbohydrate responsive element‐binding protein) transcription factor mediates activation of several regulatory enzymes of glycolysis and lipogenesis including triglyceride and fatty acid synthesis genes.

* Transcription factors <45 kDa are transported to the nucleus independently of importin/karyopherin alpha‐ and beta‐based pathways (see reference ^27^). Some of them are essential to cell survival. For example, transcription factor SFRS9 (serine/arginine‐rich splicing factor 9), also known as SRp30c or SRSF9 (≈27 kDa) is responsible for expression of >150 genes, including 50 that encode other transcription factors (see text for more details).

**Figure 1.**
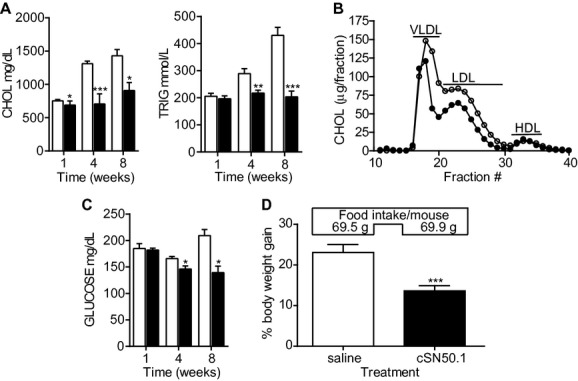
NTM reduces hyperlipidemia, hyperglycemia, and weight gain in *ldlr*^−/−^ mice fed a Western diet. At indicated times, plasma concentrations of cholesterol, triglycerides, and glucose were determined in mice fed a Western diet and treated with cSN50.1 peptide or saline by osmotic pump (A, C, D) or twice daily intraperitoneal injection (B). Mice were fasted overnight before blood collection. Results obtained at different time points represented separate groups of animals. A through C, Saline (white bars), cSN50.1 (black bars). A, Plasma concentrations of cholesterol and triglycerides at 1 week (n=10/group), 4 weeks (n=20/group), and 8 weeks (n=5/group). B, FPLC profile of plasma lipoproteins from *ldlr*^−/−^ mice at 8 weeks (n=5/group). C, Glucose concentrations in plasma at 1 week (n=5/group), 4 weeks (n=20/group), and 8 weeks (n=5/group). D, Body weight gain (%) and total food intake (g/mouse) after 4 weeks (saline, n=10; peptide, n=20) of a Western diet. Shown are mean+SEM (*P<0.05, **P<0.005 and ***P<0.0005 by Mann–Whitney test). NTM indicates nuclear transport modifier; *ldlr*^*−/−*^, low‐density lipoprotein receptor deficient; CHOL, cholesterol; TRIG, triglycerides; FPLC, fast protein liquid chromatography; LDL, low‐density lipoprotein; HDL, high‐density lipoprotein; VLDL, very low‐density lipoprotein.

Following treatment with NTM, plasma cholesterol and triglycerides levels were significantly lower in *ldlr*^−/−^ mice fed a Western diet compared with the control groups. The mean cholesterol level was 36% lower in mice treated with NTM (*P*<0.05) compared with the saline‐treated control group, in which it reached almost 1500 mg/dL after 8 weeks of Western diet (Figure 1A). Plasma triglycerides remained at a steady level in NTM‐treated mice, whereas triglyceride levels almost doubled after 8 weeks in saline‐treated control mice (Figure 1A), resulting in 53% lower levels in NTM‐treated mice (*P*<0.0005). Results from mice treated with control peptide cN50 in a pilot experiment did not differ from those of mice given saline as a control (not shown). The reduction of plasma cholesterol in NTM‐treated mice was associated with a decrease in proatherogenic LDL and VLDL cholesterol fractions (Figure 1B). Thus, treatment with NTM partially corrected disordered lipid metabolism because of excessive dietary intake of cholesterol and fats in the background of LDL receptor deficiency. Moreover, an 8‐week treatment with NTM reduced elevated fasting blood glucose levels in Western diet–fed *ldlr*^−/−^ mice from 209 to 139 mg/dL (*P*<0.05; Figure 1C). NTM‐treated animals gained less weight than those in the control group (*P*<0.0005 at 4 weeks), although the food intake of Western diet–fed *ldlr*^−/−^ mice was the same in both groups (Figure 1D).

### Attenuation of Atherosclerosis

**Figure 2.**
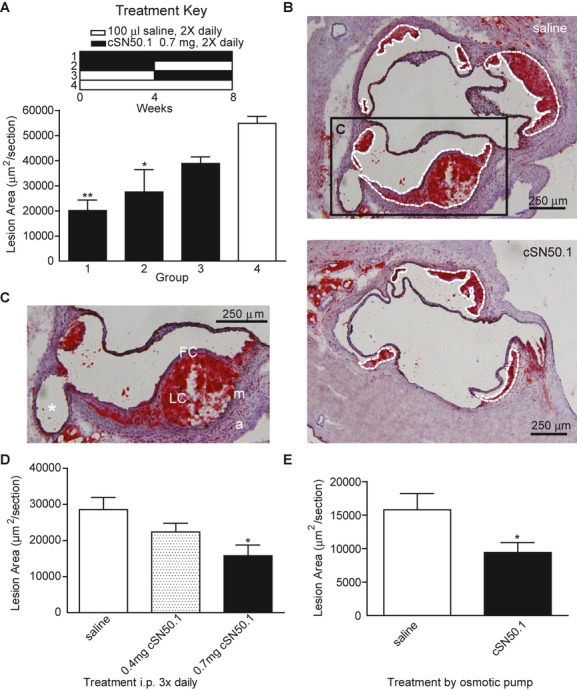
Atherosclerosis is reduced in Western diet–fed *ldlr*^*−/−*^ mice treated with NTM. A, Six‐week‐old *ldlr*^*−/−*^ mice (n=5/group) were treated with 0.7 mg/mouse cSN50.1 by intraperitoneal injection for 8 weeks twice daily (group 1) or for either the first 4 weeks (group 2) or last 4 weeks (group 3). Control mice were given an equivalent volume of saline twice daily for the entire 8 weeks (group 4). See treatment key in (A). Average lesion area was determined by Oil‐red‐O analysis of the aortic sinus (*P<0.05 and **P<0.005 by Kruskal–Wallis with a Dunn's multiple‐comparison posttest). B, Representative sections of the aortic sinus stained with Oil‐red‐O to detect neutral lipids. Sections are from mice treated for 8 weeks with saline (group 4) or cSN50.1 (group 1) while being fed a Western diet. White dashed lines depict the area used to quantify lesion size. As illustrated, lesion area was not measured past the internal elastic lamina, and the tunica media or adventitia was not included. Inset box C delineates the area magnified to show relevant structures in the plaque. C, The ostia to a coronary artery (*), lipid core (LC), fibrous cap (FC), tunica media (m) and tunica adventitia (a) are identified. Scale bar represents 250 μm in (B) and (C). D, Reduction of atherosclerosis in Western diet–fed *ldlr*^*−/−*^ mice treated with NTM was dose dependent. Mice receiving 0.7 mg/mouse of cSN50 peptide 3 times daily by intraperitoneal injection for 4 weeks (n=5/group) had a significant reduction in atherosclerotic lesion size compared with the saline‐treated group. Mice that received 0.4 mg/mouse 3 times daily were not protected from atherosclerosis (*P<0.05 by Kruskal–Wallis with a Dunn's multiple‐comparison posttest). E, Six‐week‐old *ldlr*^*−/−*^ mice treated with saline (n=11) or cSN50.1 (n=17) delivered by osmotic pump for 4 weeks (*P<0.05 by Mann–Whitney test). Shown are mean+SEM in (A), (D), and (E). NTM indicates nuclear transport modifier; *ldlr*^*−/−*^, low‐density lipoprotein receptor deficient.

We also assessed the impact of NTM treatment on the development of atherosclerosis in Western diet–fed *ldlr*^−/−^ mice. Initiation and progression of atherosclerosis were analyzed in the aortic roots of mice by Oil‐Red‐O imaging. Parallel with the suppression of plasma cholesterol and triglyceride increases (Figure 1A), *ldlr*^−/−^ mice displayed a 63% reduction in atherosclerotic lesions following an 8‐week treatment regimen of twice‐daily intraperitoneal injections of cSN50.1 peptide (0.7 mg/mouse; group 1 in Figure 2A), compared with saline‐treated controls (group 4, *P*<0.005). Another control group treated with cN50.1 control peptide in a pilot experiment had lesions similar to those in saline‐treated mice (not shown). NTM‐induced reduction of atherosclerotic lesions was documented by histological examination of aortic roots (Figure 2B and 2C). The atherosclerosis‐suppressing effect of NTM was concentration‐ and time‐dependent, as mice receiving a lower dose of cSN50.1 (0.4 mg) were not protected (Figure 2D). Remarkably, NTM treatment in either the first 4 weeks or the last 4 weeks of the Western diet protocol reduced atherosclerotic lesions by ≈50% (*P*<0.05) or ≈30%, respectively (Figure 2A). The latter result indicates the potential for NTM to arrest formation of atherosclerotic lesions. An alternative route of NTM administration via subcutaneous delivery from osmotic pumps changed weekly for 4 weeks, as described in the Methods section, also significantly suppressed development of early lesions (*P*<0.05) (Figure 2E). Cumulatively, NTM treatment of hypercholesterolemic *ldlr*^*−/−*^ mice was strikingly effective in a dose‐ and time‐dependent manner, irrespective of delivery route, to prevent the initiation and reduce the progression of atherosclerosis. Analysis of blood cell populations and blood chemistries did not reveal any significant changes (other than those shown) in mice given NTM, compared with control animals, after 8 weeks of treatment, and there were no overt signs of surgical wound infection, delayed healing, or illness in treated animals.

### NTM Treatment Prevents Accumulation of Cholesterol and Triglycerides in the Liver and Reduces Markers of Liver Inflammation

**Figure 3.**
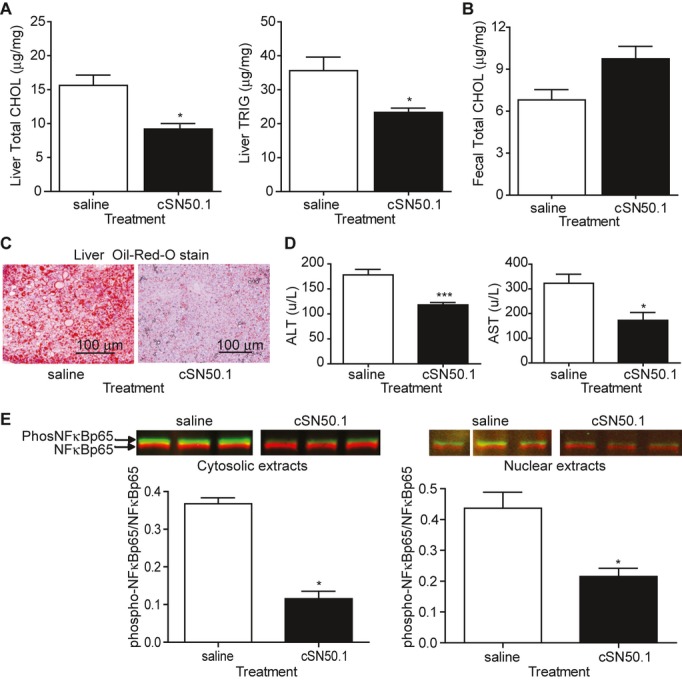
Lipid accumulation and markers of inflammation are reduced in the livers of NTM‐treated mice. Groups of *ldlr*^*−/−*^ mice (n=5/group) were fed a Western diet and treated with NTM or saline control by osmotic pump for 8 weeks. Liver cholesterol and triglyceride concentrations were measured as well as cholesterol content of feces. Histological sections of liver were analyzed for lipid content, and NFκB RelA (p65) phosphorylation in cytosolic and nuclear liver extracts was analyzed by immunoblotting. Plasma concentrations of ALT and AST were determined after 4 wks. Results obtained at different time points represented separate groups of animals. A, Liver concentrations of cholesterol and triglycerides. B, Fecal cholesterol. C, Liver sections stained with Oil‐red‐O for neutral lipids. D, Liver transaminases ALT and AST in plasma. E, Immunoblots of cytosolic and nuclear extracts from control (saline) and cSN50.1‐treated mice. Shown are 3 of 5 samples for each saline‐ and cSN50.1‐treated group. Saline control lanes were spliced to remove a damaged area of the membrane, but all lanes are from the same blot for each cytosolic and nuclear extracts. Quantitative analysis of immunoblots indicate significant suppression of phosphorylated NFκB RelA (green) expressed as its relative ratio to total RelA (red) in both cytosolic and nuclear extracts. Shown are mean+SEM (*P<0.05 and ***P<0.0005 by Mann–Whitney test). NTM indicates nuclear transport modifier; *ldlr*^*−/−*^, low‐density lipoprotein receptor deficient; CHOL, cholesterol; TRIG, triglycerides; ALT, alanine transaminase; AST, aspartate transaminase; NF‐κB, nuclear factor kappa–light‐chain enhancer of activated B cells.

In parallel with the precipitous decline in plasma cholesterol and triglyceride levels, NTM significantly reduced the abnormal accumulation of cholesterol and triglycerides in the liver by 41% (*P*<0.05) and 34% (*P*<0.05), respectively (Figure 3A) after 8 weeks while intestinal disposal of cholesterol was increased by 32% (Figure 3B). Consequently, overall fat content in the liver was dramatically reduced (Figure 3C), although mice in both groups consumed the same amount of high‐fat‐ and high‐cholesterol‐containing food (Figure 1D).

NTM also significantly reduced elevated liver alanine and aspartate transaminases (ALT and AST) in Western diet–fed *ldlr*^−/−^ mice (Figure 3D), possibly by attenuating the liver inflammatory response to growing fat content. Similar to saline‐treated controls, animals receiving control cN50 peptide in a pilot experiment displayed elevated levels of liver transaminases (not shown). These biomarker changes are indicators of attenuated steatohepatitis^30^ and were also significantly reduced by NTM in other models of liver inflammation.^29^ Therefore, we analyzed the fatty livers of Western diet–fed *ldlr*^−/−^ mice for activation and nuclear transport of NFκB RelA (p65). Western diet–fed *ldlr*^−/−^ mice treated with saline displayed the active, phosphorylated form of NFκB RelA (p65) in cytosolic and nuclear fractions of liver cells whereas it was significantly reduced in the livers of NTM‐treated mice (Figure 3E). The results shown in Figures 1 and 3 indicate that NTM potentially possessed dual functions, not only inhibition of nuclear transport of proinflammatory SRTFs^5,26^ responsible for the inflammatory response, but also inhibition of nuclear transport of transcription factors responsible for hyperlipidemia. Therefore, we embarked on defining the mechanism of NTM action in reference to nuclear import of transcription factors responsible for lipid homeostasis such as SREBPs.

### NTM Binds to Importin Beta, the Nuclear Transport Shuttle for SREBPs, and Inhibits Their Nuclear Import

**Figure 4.**
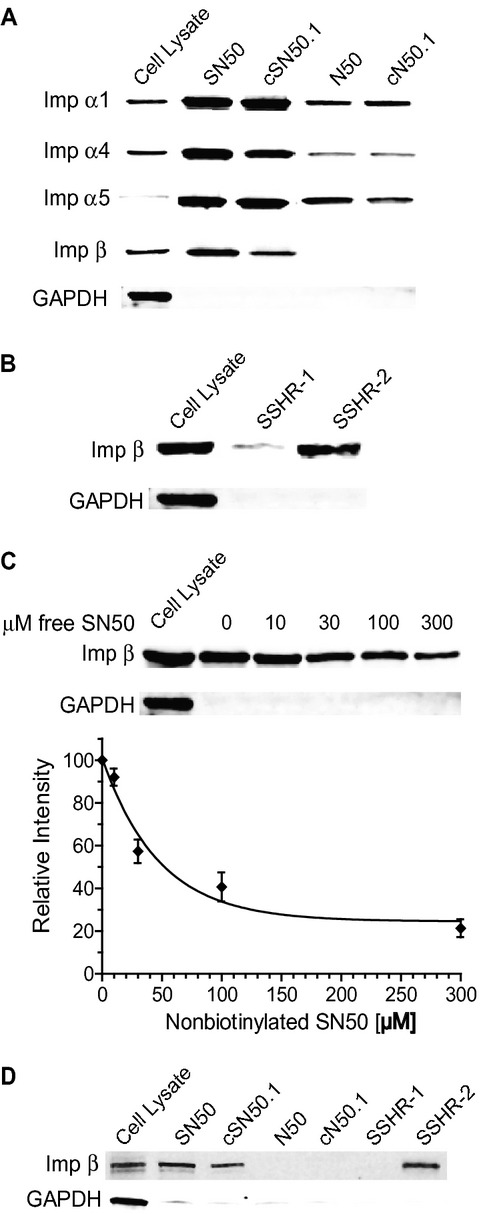
Binding of NTM peptides and peptide modules to importins alpha and beta. A, Importins (Imp) pull‐down assay shows interactions between NTM peptides (linear SN50 and cyclized cSN50.1) and their non‐cell‐penetrating NLS modules (N50 and cN50.1) with nuclear transport adaptors importins alpha and beta using whole‐cell extracts from Jurkat T cells. GAPDH was used as a nonspecific cellular protein control. B, Pull‐down assay with the 2 parts of the NTM hydrophobic region, SSHR‐1 and SSHR‐2, indicating that SSHR‐2 binds to importin beta. C, Nonbiotinylated NTM inhibited binding of importin beta to immobilized SN50 in a concentration‐dependent manner in a competition binding assay, shown as the mean±SD of 3 independent experiments. D, Importin beta binds to the SSHR‐2 region of NTM peptides in a pull‐down assay using HepG2 whole‐cell extracts. Blots in (A) through (D) are representative of ≥3 independent experiments, and all lanes shown in each row are from the same membrane for each primary antibody. In panel A, a representative GAPDH control immunoblot is shown. In all other panels the same membrane was blotted for GAPDH. NTM indicates nuclear transport modifier; NLS, nuclear localization sequence; SSHR, signal sequence hydrophobic region.

As a first step in exploring the potential mechanism of NTM‐induced lipid homeostasis we determined whether NTM interacts with the nuclear transport shuttle, importin beta, the sole nuclear transport adaptor protein responsible for translocating nSREBPs to the nucleus.^13^ Table 1 shows the full‐length NTM peptides and their constitutive peptide modules that we employed in pull‐down assays to identify their binding partners. We used human Jurkat T cells to analyze the interaction of NTM with importins alpha and beta because T lymphocytes are involved in hepatitis and vascular inflammation and our initial study of nuclear transport mediated by importin alpha was performed in these cells.^5^ As documented in Figure 4A, the full‐length biotinylated NTMs (SN50 and cSN50.1) interacted with importins alpha 1, 4, and 5 but not with the nonspecific cytoplasmic control protein GAPDH. Surprisingly, the full‐length peptides also interacted with importin beta (Figure 4A). In contrast, biotinylated N50 and cN50.1 peptides, which reproduce the basic NLS derived from NFκB1 (p50) but do not contain an SSHR sequence, did not react with importin beta but did maintain reactivity with importins alpha 1, 4, and 5. A similar binding pattern was recorded by us with cell extracts from human HeLa cells (not shown). Because N50 and cN50.1 peptides lack an SSHR, we inferred that this motif provided a second binding function to NTM, namely, interaction through its hydrophobic residues with importin beta. We localized the importin beta‐binding function to the SSHR‐2 that adjoins the NLS (Table 1 and Figure 4B). Binding to importin beta was reversible because free, nonbiotinylated SN50 peptide competitively inhibited biotinylated SN50 peptide binding to importin beta (Figure 4C). As documented in Figure 4D, we verified that NTMs (SN50 and cSN50.1) interacted with importin beta in human liver‐derived hepatoma HepG2 cell extracts in the same manner, but not with the control protein, GAPDH. This second function of NTM indicated that it might modulate nuclear transport of SREBPs that bear a bHLH domain recognized by importin beta but not importins alpha.

**Figure 5.**
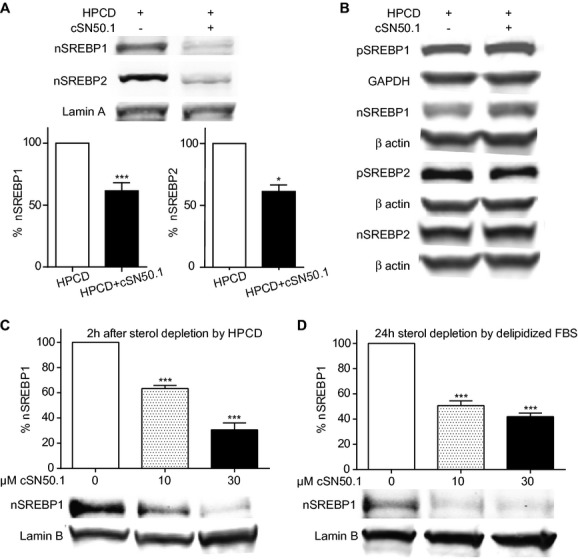
NTM inhibits nuclear translocation of SREBPs in sterol‐depleted HepG2 cells. Nuclear and cytosolic extracts were immunoblotted with antibodies to SREBP1 and SREBP2. For normalization, nuclear extracts were also immunoblotted with anti‐Lamin A/C or Lamin B, whereas cytosolic extracts were normalized to GAPDH or β actin immunoblot controls as indicated. All lanes shown in each row are from the same membrane for each primary antibody, and the same membrane was blotted for the appropriate loading control protein unless otherwise indicated. Values from sterol‐depleted samples without cSN50.1 treatment were set to 100%, and percent values for peptide‐treated samples were calculated. The difference represents percent inhibition of nuclear translocation by cSN50.1 shown as the mean±SEM of ≥3 independent experiments. A and B, Cells incubated with hydroxypropyl‐β‐cyclodextrin (HPCD) for 15 minutes, then treated with 0 or 30 μmol/L cSN50.1 in DMEM+5% delipidized FBS for 2 hours. A, Nuclear extracts (*P<0.05 and ***P<0.0005 by Student's *t* test). Shown is a representative Lamin A control immunoblot. B, Cytosolic extracts. Statistical analyses of values from cytosolic extracts in 4 experiments (SREBP1) or 3 experiments (SREBP2) showed no significant differences (by Student's *t* test) between precursor (pSREBPs) outside the nucleus. A significant increase of mature, cleaved SREBP1 (nSREBP1) is apparent in the same cytosolic extracts (*P*<0.05 by Student's *t* test), though cytosolic nSREBP2 is not significantly increased. C and D, Concentration‐dependent inhibition of SREBP1 normalized to Lamin in sterol‐depleted HepG2 cells. C, Rapid depletion of sterols with HPCD as in (A). D, Gradual depletion of sterols in cells treated with 0, 10, or 30 μmol/L cSN50.1 in DMEM+5% delipidized FBS only for 24 hours (***P<0.0005 by 1‐way ANOVA with a Tukey's multiple‐comparison posttest). NTM indicates nuclear transport modifier; SREBP, sterol regulatory element‐binding protein; FBS, fetal bovine serum; ANOVA, analysis of variance.

We tested this possibility by examining the effect of NTM on nuclear transport of SREBPs in sterol‐depleted HepG2 cells. In response to a deceased intracellular level of sterols, their sensor Scap initiates translocation of ER membrane‐bound SREBPs to the Golgi apparatus, where S1P and S2P proteases split off the mature nuclear forms that are recognized in the cytoplasm by importin beta and transported to the nucleus.^13^ Treatment with cSN50.1 for 2 hours prevented this last step, thereby reducing nuclear accumulation of nSREBP1 and nSREBP2 (Figure 5A). We addressed the effect of NTM on earlier steps in SREBP expression and processing by also examining SREBPs in the cytosolic fraction. Precursor forms of SREBP1 and SREBP2 (pSREBP1 and pSREBP2) remained unchanged at the same 2‐hour point in NTM‐treated cells as in the control. Moreover, processing of precursor forms of SREBPs to their nuclear (mature) forms was also unaffected by NTM as documented by their presence in the cytosolic fraction (Figure 5B). In fact, there was significantly more nSREBP1 in the cytosolic fraction of cells treated with cSN50.1 after depletion of lipids by HPCD (Figure 5B, *P*<0.05, quantitative graph not shown). The attenuating effect of NTM on nuclear translocation of SREBP1 was demonstrated in 2 intracellular sterol‐depleting conditions (Figure 5C and 5D). After using HPCD to rapidly deplete cells of sterols, the effect of cSN50.1 was concentration dependent. When sterols were gradually depleted by prolonged incubation in medium containing delipidized FBS, maximum reduction of the nuclear pool of nSREBP1 was achieved with 10 μmol/L cSN50.1, whereas a higher concentration (30 μmol/L) did not further reduce nSREBP1 in the nucleus by a significant amount. Notably, NTM did not completely reduce nuclear transport of SREBPs in either protocol. Thus, under the experimental conditions of our studies, inhibition of SREBP nuclear transport was incomplete, allowing basal expression of SREBP‐regulated genes. Nevertheless, these studies support the newly discovered second function of NTM, namely, time‐ and concentration‐dependent attenuation of importin beta‐mediated nuclear transport of nSREBPs. We next asked the question: does NTM treatment reduce SREBP expression and action in the liver?

### In Vivo Reduction of SREBPs and Their Target Genes That Encode Cholesterol‐, Triglyceride‐, and Fatty Acid–Synthesizing Enzymes and Cholesterol Uptake Proteins

**Figure 6.**
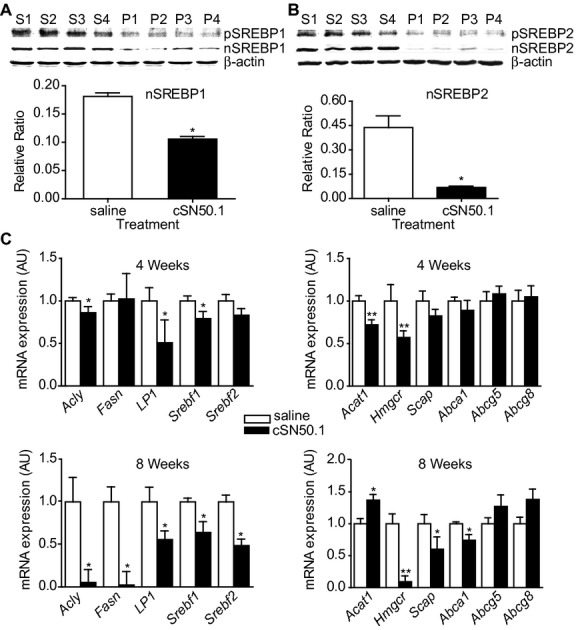
NTM treatment attenuates expression of the master regulators of lipid metabolism in the liver. SREBP transcription factors and their target gene transcripts in *ldlr*^−/−^ mice fed a Western diet. A and B, Immunoblot analysis of (A) SREBP1 and (B) SREBP2 proteins in whole‐cell liver extracts from mice treated with saline (S1 to S4) or cSN50.1 peptide (P1 to P4) for 8 weeks by osmotic pump. Full‐length precursor (pSREBP) and mature nuclear (nSREBP) forms are present in liver extracts from control mice but are suppressed in extracts from NTM‐treated mice. Quantitative analysis of immunoblots indicates significant suppression of nSREBP1 and nSREBP2 expressed as their relative ratio to beta actin control. Each panel shows samples from the same membrane. C, Transcript expression of SREBP1 and SREBP2 and their target genes in the livers of mice treated with NTM administered by osmotic pump for 4 and 8 weeks with saline (white bars) or cSN50.1 peptide (black bars). Expression was measured by real‐time RT‐PCR with 18S mRNA used as an invariant control. Shown are mean+SEM of 5 mice per group (*P<0.05 and **P<0.005 by Mann–Whitney test). NTM indicates nuclear transport modifier; *ldlr*^*−/−*^, low‐density lipoprotein receptor deficient; SREBP, sterol regulatory element‐binding protein; RT‐PCR, reverse‐transcription polymerase chain reaction.

**Figure 7.**
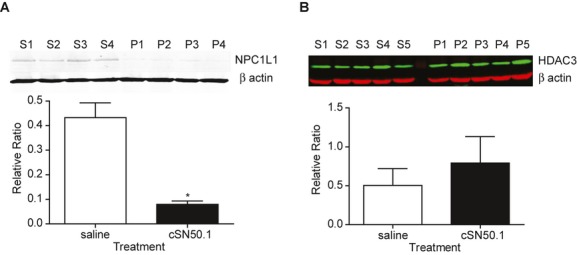
NTM suppresses Niemann‐Pick C1‐like 1 (NPC1L1) protein expression in the liver, whereas nuclear transport of histone deacetylase 3 (HDAC3) protein is not reduced. Immunoblots of liver extracts from *ldlr*^*−/−*^ mice fed a Western diet and treated with saline control (S) or cSN50.1 peptide (P) by osmotic pump for 8 weeks. Each panel shows samples from the same membrane. A, NPC1L1 in whole‐cell liver extracts. Quantitative analysis of immunoblot indicates significant suppression of NPC1L1 protein in NTM‐treated animals expressed as its relative ratio to β actin loading control. Shown are the mean+SEM of 4 mice/group (*P<0.05 by Mann–Whitney test). B, HDAC3 in liver nuclear extracts. Quantitative analysis of immunoblot indicates no significant difference (by Mann–Whitney test) between HDAC3 protein in saline versus cSN50.1 peptide‐treated mice. HDAC3 protein (green) is expressed as its relative ratio to β actin loading control (red). Shown are the mean+SD of 5 mice/group. NTM indicates nuclear transport modifier; *ldlr*^*−/−*^, low‐density lipoprotein receptor deficient.

As the liver is the main organ in which SREBP transcription factors act as the master regulators of lipid homeostasis,^7–9^ we examined their expression in liver extracts from Western diet–fed *ldlr*^−/−^ mice treated for 4 and 8 weeks with NTM or saline (control). In contrast to the short‐term (2‐hour) experiment with cultured HepG2 cells (see above), both precursor and nuclear forms of SREBP1 and SREBP2 proteins were reduced in NTM‐treated *ldlr*^−/−^ mice compared with saline‐treated controls (*P*<0.05; Figure 6A and 6B). This reduction in SREBP protein expression was paralleled by progressively reduced transcripts for both SREBP1 (*srebf1*) and SREBP2 (*srebf2*) (*P*<0.05 for *srebf1* after 4 weeks; *P*<0.05 for both after 8 weeks) in the livers of NTM‐treated mice (Figure 6C). These changes in SREBP1 and SREBP2 expression over time mirror the suppression of diet‐induced plasma cholesterol and triglyceride levels (Figure 1A). SREBPs not only regulate their own cognate genes through an autoregulatory feed‐forward loop but are also known to regulate expression of >30 other genes that encode a cascade of cholesterol and fatty acid synthesis enzymes and cholesterol uptake proteins.^7^ Therefore, we analyzed liver RNA for transcripts for some of these other genes. We noted progressive reduction of mRNA for HMG‐CoA reductase (*hmgcr*), the rate‐controlling enzyme in cholesterol synthesis and a target of statins^31^ (Figure 6C), reaching significantly decreased expression (*P*<0.005) after 4 weeks and 8 weeks of treatment compared with saline‐treated controls. Likewise, representative transcripts encoding other SREBP‐controlled genes, ATP citrate lyase (*acly*) and fatty acid synthase‐1 (*fasn1*)*,* were significantly reduced (*P*<0.05 for both) after 8 weeks of NTM treatment. The latter gene is regulated by SREBP1c, a dominant isoform in human and mouse livers.^32^ Thus, NTM attenuated not only expression of SREBP transcription factors but also their target genes involved in cholesterol, triglyceride, and fatty acid synthesis.^7^ Importantly, genes encoding proteins responsible for cholesterol enterohepatic efflux, ABCG5 (*abcg5*) and ABCG8 (*abcg8*), were not reduced (Figure 6C). In contrast, we noted significant NTM‐induced reduction of SREBPs‐regulated Niemann‐Pick C1‐like 1 (NPC1L1) protein (*P*<0.05; Figure 7A), a key enterohepatic cholesterol absorption receptor.^33^ This reduction of NPC1L1 in NTM‐treated mice is consistent with the reduced accumulation of lipids in their livers and increased cholesterol clearance in feces (Figure 3A and B). As a comparative control for nuclear transport, we also analyzed expression of the karyophilic protein histone deacetylase 3 (HDAC3).^34^ HDAC3 nuclear content was not significantly changed in NTM‐treated *ldlr*^−/−^ mice fed a Western diet for 8 weeks, indicating that NTM did not modulate its nuclear transport (Figure 7B).

### NTM Reduces Nuclear Import of Carbohydrate Response Element‐Binding Protein (ChREBP)

**Figure 8.**
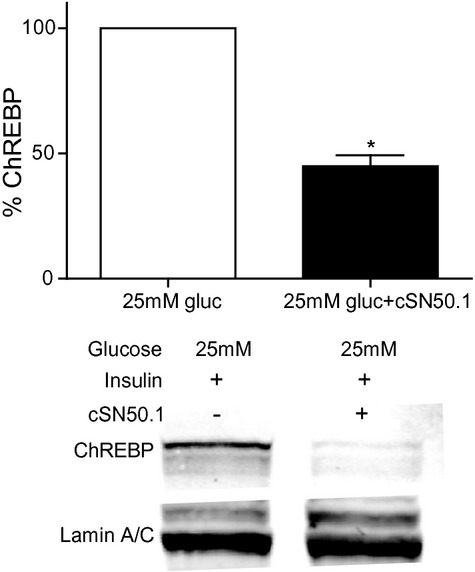
NTM inhibits glucose‐induced nuclear translocation of ChREBP in HepG2 cells. HepG2 cells were starved for 24 hours in DMEM containing 5.5 mmol/L glucose then refed with 25 mmol/L glucose+100 nmol/L insulin±30 μmol/L cSN50.1 for 24 hours. Nuclear extracts were immunoblotted with antibodies to ChREBP and Lamin A/C (loading control). Samples shown are from the same membrane. Values from glucose‐induced samples without cSN50.1 treatment were set to 100% and percent values for peptide‐treated samples calculated. The difference represents percent inhibition of nuclear translocation by cSN50.1, shown as the mean+SEM of 3 independent experiments (*P<0.05 by Student's *t* test). NTM indicates nuclear transport modifier; ChREBP, carbohydrate responsive element‐binding protein.

We tested the effect of NTM on glucose‐induced nuclear transport of ChREBP in HepG2 cells to explore a potential mechanism for the normalization of fasting glucose levels by NTM in Western diet–fed *ldlr*^−/−^ mice (Figure 1C). ChREBP is a glucose‐activated transcription factor that regulates expression of genes involved in glycolysis, lipogenesis, and gluconeogenesis responsible for converting excess carbohydrates into triglycerides rather than glycogen.^35^ HepG2 cells were first starved in low glucose medium (5.5 mmol/L), then refed with high glucose medium (25 mmol/L) and insulin. As shown in Figure 8, NTM (cSN50.1 at 30 μmol/L) attenuated nuclear accumulation of ChREBP by ≈50%.

## Discussion

These results establish the validity of targeting nuclear transport to reduce hypercholesterolemia, hypertriglyceridemia, atherosclerosis, and fatty liver in a murine model of LDL receptor deficiency. The salient findings in support of targeting nuclear transport include: (1) application and characterization of a bifunctional NTM that attenuated synthetic pathways for cholesterol, triglyceride, and fatty acid production in *ldlr*^*−/−*^ mice fed a Western diet; (2) NTM attenuation of initiation and progression of atherosclerosis, which exemplifies vascular inflammation evoked by metabolic insults; (3) concomitant prevention of fatty liver development and reduction of inflammatory biomarkers of hepatocellular injury, a hallmark of steatohepatitis; and (4) less weight gain in NTM‐treated animals, although food intake remained unchanged. We also observed a reduction in fasting mouse blood hyperglycemia in response to NTM treatment. Thus, by modulating nuclear transport of SREBPs, ChREBP, and SRTFs, a new strategy for comprehensive reduction of metabolic inflammation has emerged.

Metabolic inflammation from accumulation of noxious levels of lipids affects the vascular system, leading to atherosclerosis. When fed a high‐fat Western diet, *ldlr*^*−/−*^ mice develop atherosclerosis mimicking that in human familial hypercholesterolemia, in which genetic defects in LDL receptor function increase the risk of early heart attack >10‐fold.^14,36^ Atherosclerotic lesions are formed by the accumulation of cholesterol‐laden macrophages in the aortic wall. The reduction in these lesions by NTM treatment is most likely associated with reduced plasma cholesterol and triglyceride levels, an improved atherogenic lipoprotein profile, and reduced SRTF‐mediated proinflammatory signaling in macrophages and other immune cells.

Several mediators that participate in this atherosclerosis‐promoting vascular inflammatory process are regulated by nuclear transport of NFκB and other SRTFs such as AP‐1, NFAT, and STAT1. SRTFs are widely distributed in the vascular and immune cells that gather in atherosclerotic lesions.^37–40^ Cholesterol crystals are found in lesions in the earliest stages of diet‐induced atherogenesis, together with the appearance of immune cells.^41^ The formation of cholesterol crystals in macrophages is associated with inflammasome activation, which generates mature forms of IL1β and IL18 in response to Toll‐like receptor (TLR) 2/4 signaling to the nucleus.^41–42^ The SRTF Nrf2 is essential for inflammasome activation and exacerbates atherosclerosis without altering lipid metabolism.^43–44^ Development of atherosclerotic lesions also depends on genes encoding the innate immunity receptors TLRs 2 and 4, as well as their pivotal adapter MyD88.^45–47^ These signaling pathways are attenuated by NTM, which targets the nuclear transport checkpoints for importins alpha and beta strategically positioned downstream of Toll‐like receptors and MyD88. Thus, nuclear transport serves as a common nexus in the innate and adaptive immunity pathways that signal to the nucleus.^28^ In addition, NTM partially restores lipid homeostasis by reducing nuclear transport of SREBPs. SREBP‐1a, SREBP‐1c, and SREBP2 regulate expression of multiple genes encoding cholesterol and triglyceride synthesizing enzymes and binding proteins.^7–9^

Significantly, NTM does not modulate all nuclear transport. For example, we observed that expression of *abcg5* and *abcg8* genes encoding proteins responsible for cholesterol enterohepatic efflux was not altered in the livers of NTM‐treated mice (Figure 6). Likewise, NTM did not modulate nuclear translocation of a 50 kDa karyophilic protein essential for cell viability, NAD‐dependent HDAC3 (Figure 7B).^34^ Expression of HDAC3 (and HDAC1) depends on serine/arginine‐rich splicing factor 9 (SFRS9, also known as SRp30c or SRSF9).^48^ Notably, this low‐molecular‐weight (≈27 kDa) transcription factor is responsible for expression of >150 genes, including 50 that encode other transcription factors.^#^ Many small transcription factors (<45 kDa) are essential to cell survival and freely cross the nuclear membrane without assistance from the nuclear transport adaptor proteins targeted by NTM.^27^ Therefore, it is unlikely that NTM is involved in their nucleocytoplasmic trafficking.

It is also noteworthy that concentration‐ and time‐dependent inhibition of nuclear transport of SREBPs was incomplete, allowing partial nuclear transport of these transcription factors even in the presence of NTM (Figure 5). Partial nuclear translocation of SREBPS is consistent with the reversible nature of NTM binding to importin beta (Figure 4C). This aspect of NTM action coupled to its relatively short intracellular persistence (≈180 minutes)^49^ suggests that NTM modulation of nuclear transport of other members of the bHLH‐Zip family, such as ChREBP, may also be short‐lived and reversible. As with SREBPs, incomplete suppression of ChREBP nuclear translocation was observed in HepG2 cells (Figure 8), which could potentially allow basal expression of genes regulated by ChREBP. Further work will be required to explore these new aspects of NTM action more fully.

The examples enumerated above mitigate the concern that broad, albeit partial, inhibition of nuclear transport could be undesirable for fundamental cell function. These considerations are further supported by the apparent lack of toxicity of NTM administration, as evidenced by normal blood chemistries and no changes in red blood cells, white blood cells, major lymphocytes subsets, or blood platelets in *ldlr*^*−/−*^ mice fed a Western diet. When mice in this study were administered NTM or saline by subcutaneous osmotic pumps surgically implanted and replaced each week for 8 weeks, they did not display any apparent signs of abnormal wound healing, infection, or general susceptibility to environmental pathogens, and no other side effects were observed. Contrary to the conventional assumption that using NTM to target nuclear transport of SRTFs, the mainstays of innate immunity and inflammation,^28^ would compromise host defenses, NTM improved the outcome of polymicrobial sepsis and pulmonary anthrax when added to ineffective antimicrobial therapy.^15,50^

It is of note that in a study of Type 1 diabetes (T1D) in NOD mice, intense, short‐term treatment with NTM eliminated autoreactive B and T cells infiltrating pancreatic islets, resulting in long‐term (1 year) suppression of diabetes in this relevant model for human autoimmune disease.^20^ This suppression is remarkable as the intracellular persistence of NTM is approx. 180 min. with an estimated intracellular half‐life of 90 minutes.^49^ Furthermore, preventing or reversing autoimmune destruction of islets may reduce hyperlipidemia and accelerated atherosclerosis, known complications of T1D.^51^

Steady accumulation of noxious levels of lipids is responsible for progression of fatty liver into steatohepatitis,^30^ and targeting individual SREBPs is not sufficient to avert it. Selective knockout of the *srebpf1* gene does not reduce the content of cholesterol and triglycerides in the livers of Western diet–fed *ldlr*^−/−^ mice, although, significantly, these mice do display reduced plasma cholesterol and triglyceride levels, improving the atherogenic lipoprotein profile and reducing atherosclerosis.^12^ In contrast, in *ob/ob* mice, genetic ablation of Scap, which senses intracellular sterols and controls processing of all 3 precursor SREBPs into their nuclear forms, did prevent fatty liver.^52^ However, obesity, hyperinsulinemia, and hyperglycemia persisted. Furthermore, silencing Scap with siRNA reduced all 3 nuclear SREBPs, preventing fatty liver and hypertriglyceridemia in sucrose‐fed hamsters.^52^ In our study, NTM attenuated not only fatty liver but also its attendant inflammation (steatohepatitis) by normalizing its inflammatory biomarkers (elevated levels of the liver transaminases ALT and AST in plasma) and reducing phosphorylated NFκB RelA (p65) in liver cell nuclei (Figure 3D and 3E).

**Figure 9.**
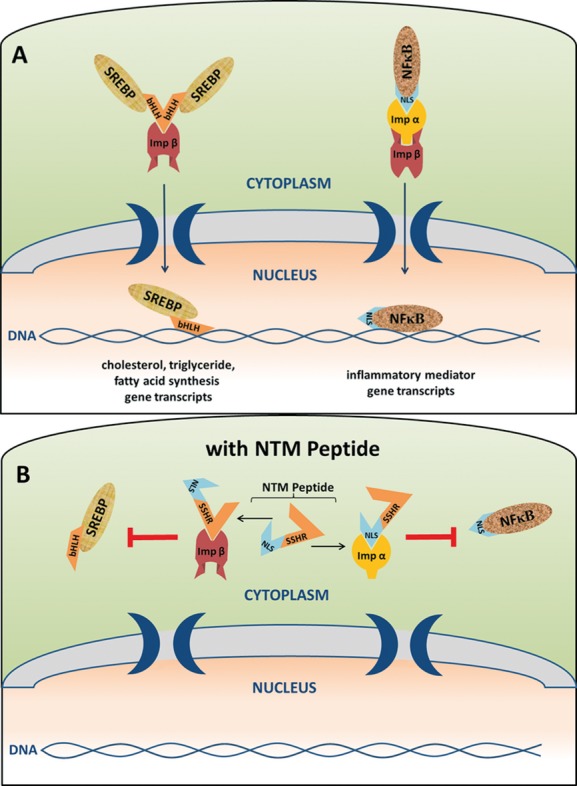
Two pathways of nuclear import and their inhibition by a bifunctional NTM. A, Nuclear translocation of transcription factors that bear a basic bHLH‐Zip motif (bHLH), such as the SREBP proteins, is mediated by binding of their bHLH region directly to importin beta (Imp β, left). Consequently, multiple genes that encode proteins involved in cholesterol, triglyceride, and fatty acid synthesis are activated. Transcription factors containing a classic NLS motif, such as NFκB, are ferried by binding of their NLS region to importin alpha (Imp α, right), which then forms a complex with importin beta for nuclear translocation to activate a myriad of genes that encode mediators of inflammation and immunity. B, In cells treated with NTM peptide, the SSHR‐2 domain of the peptide occupies the bHLH‐Zip docking site on importin beta (left), whereas the NLS domain occupies the NLS‐binding pocket on importin alpha (right), preventing transcription factor attachment and subsequent nuclear translocation and gene transcription. NTM indicates nuclear transport modifier; SREBP, sterol regulatory element‐binding protein; NLS, nuclear localization sequence; SSHR, signal sequence hydrophobic region.

Thus, bifunctional NTM can attenuate transport of both SRTFs and SREBPs, as documented in this study and depicted schematically in Figure 9. As SREBPs lack a classic NLS, their nuclear translocation is solely mediated by association of their highly conserved bHLH‐Zip region dimers with importin beta (Figure 9A, left) through mostly hydrophobic interactions.^13,53^ In contrast, proinflammatory SRTFs, exemplified by NFκB, bear an NLS that forms a complex with its cognate importin/karyopherin alpha that then uses importin beta through a different binding site for docking to the nuclear pore (Figure 9A, right).^13^ Our studies document that NTM displays dual functions: (1) inhibition of nuclear transport of SREBPs with subsequent attenuation of their target genes responsible for hyperlipidemia (Figure 9B, left); and (2) reduced nuclear transport of proinflammatory SRTFs exemplified by NFκB, which controls the inflammatory response to metabolic insults (Figure 9B*,* right). Moreover, these 2 functions of NTM may work in concert. Although ChREBP is a bHLH‐Zip domain transcription factor in the same family as SREBPs, it also contains a classic NLS recognized by importins alpha.^54^ Therefore, the importin alpha‐binding domain of cSN50.1 might also contribute to the inhibition of ChREBP nuclear transport because the importin beta site that binds the bHLH‐Zip domain on SREBPs is distinct from the point of contact between importin beta and importin alpha.^13^ Future studies will help in discerning which importin‐mediated pathway contributes most to restoration of lipid homeostasis.

Lipid homeostasis comprises a tightly regulated physiologic balance between dietary lipid intake, endogenous production, and intestinal disposal. It is thus significant that hyperlipidemia, atherosclerosis, and fatty liver, which are on a steady rise worldwide,^55^ are reduced by NTM in a mouse model of familial hypercholesterolemia without overt signs of general toxicity. Modulation of nuclear transport to reduce hyperlipidemia, the major cause of hepatobiliary and cardiovascular system complications, characterizes NTMs as prototypical members of a new class of dual‐function lipid‐lowering and anti‐inflammatory agents on the basis of the evidence presented here. In *ldlr*^−/−^ mice fed a Western diet, hyperglycemia is associated with elevated levels of triglycerides, thereby forming “the deadly combination” that leads to metabolic syndrome.^56^ Therefore, reduction of glucose and triglyceride levels by NTM, along with ameliorated weight gain, extends its action to the major facets of this syndrome.^55–56^ Taken together, our findings encourage us to embark on further studies of the dual lipid‐lowering and anti‐inflammatory actions of NTM in potential correction of metabolic syndrome and its complications.
